# Effect of Short-Term Water Exposure on the Mechanical Properties of Halloysite Nanotube-Multi Layer Graphene Reinforced Polyester Nanocomposites

**DOI:** 10.3390/polym9010027

**Published:** 2017-01-14

**Authors:** Mohd Shahneel Saharudin, Rasheed Atif, Fawad Inam

**Affiliations:** 1 Institute of Product Design and Manufacturing (UniKL IPROM), Universiti Kuala Lumpur, 56100 Cheras, Kuala Lumpur, Malaysia; 2 Department of Mechanical and Construction Engineering, Faculty of Engineering and Environment, Northumbria University, Newcastle upon Tyne NE1 8ST, UK; aatif.rasheed@northumbria.ac.uk (R.A.); fawad.inam@northumbria.ac.uk (F.I.)

**Keywords:** nanocomposites, halloysite nanotubes, multi-layer graphene, water absorption, mechanical properties

## Abstract

The influence of short-term water absorption on the mechanical properties of halloysite nanotubes-multi layer graphene reinforced polyester hybrid nanocomposites has been investigated. The addition of nano-fillers significantly increased the flexural strength, tensile strength, and impact strength in dry and wet conditions. After short-term water exposure, the maximum microhardness, tensile, flexural and impact toughness values were observed at 0.1 wt % multi-layer graphene (MLG). The microhardness increased up to 50.3%, tensile strength increased up to 40% and flexural strength increased up to 44%. Compared to dry samples, the fracture toughness and surface roughness of all types of produced nanocomposites were increased that may be attributed to the plasticization effect. Scanning electron microscopy revealed that the main failure mechanism is caused by the weakening of the nano-filler-matrix interface induced by water absorption. It was further observed that synergistic effects were not effective at a concentration of 0.1 wt % to produce considerable improvement in the mechanical properties of the produced hybrid nanocomposites.

## 1. Introduction

Unsaturated polyester (UP) resins are commonly used in thermosetting polymers due to their low price and easy processability into composite fabrications [1,2]. In general, UP resins made up of a condensation reaction between a glycol and an unsaturated dibasic acid [3]. UP resins have been used in automobile, water tanks, packaging and building materials [4,5]. Undoubtedly the thermal and mechanical properties of these polymers (unsaturated polyester) are generally improved by the addition of inorganic additives (particles and fibres reinforcements) [6,7,8]. Their effectiveness can be associated to the interfacial adhesion between the polymer matrix and the reinforcement material which typically influenced by a relative incompatibility between the organic and inorganic phases [9,10].

Polymeric materials are vulnerable to environmental degradation caused by moisture, solvents, oil, temperature, mechanical loads and radiation [11,12]. In particular, all polymer composites absorb moisture and the water molecules can act as a plasticizer by influencing simultaneously the fibres, the matrix and the interface, thus creating regions of poor transfer efficiency, which results in a reduction of mechanical properties [13,14]. Several factors are known to affect the way in which composite materials absorb water [13,15], such as temperature, fibre volume fraction, reinforcement architecture, fibre nature (permeable or impermeable), area of exposed surfaces, polarity of the molecular structure, degree of cross-linking and degree of crystallinity [14].

A wide range of engineering properties can be improved with a low level of halloysite nanotubes typically less than 5 wt % [16]. In our previous work, we studied the tensile properties of polyester nanocomposites reinforced with halloysite nanotubes. We found that the incorporation of halloysite nanotubes increased Young’s modulus up to 70% compared to unfilled polyester exposed to diluted methanol [17]. Tensile strength and impact toughness increased 17.4% and 184% respectively [17]. Other improved physical and engineering properties include fire retardancy [18,19], barrier resistance [20] and ion conductivity [16]. Another advantage of clay nanocomposites is that the optical properties of the polymer are not considerably affected. This property is very useful for medical applications where optical clarity is vital such as catheter connectors, cardiac surgery products and intravenous infusion components [21,22]. Alamri and Low studied the effect of water on the mechanical properties of halloysite nanotubes (HNT) reinforced epoxy. They observed that the incorporation of halloysite nanotubes was able to reduce water absorption and improve mechanical properties of the nanocomposites after water immersion [23].

Graphene-based polymer composites are very promising candidates for high performance materials that offer improved mechanical, thermal, gas barrier and electrical properties [24,25,26]. Graphene based materials have been used in different fields such as composites, coatings, electronics devices, energy storage, sensors and biomedical applications [24]. Atif et al. in their study reported that multi-layer graphene (MLG) improved Young’s modulus and microhardness by 25.7% and 18.3%, respectively [27]. The MLG also increased *T*_g_ and storage modulus compared to unfilled epoxy [28,29,30].

In this work, the effect of short term water absorption on the mechanical properties of polyester based nanocomposites reinforced with halloysite nanotubes (HNT), multi-layer graphene (MLG) and HNT-MLG (hybrid fillers) has been studied. The influence of HNT, MLG and HNT-MLG has been tested in terms of weight gain of nanocomposites due to water absorption. The effect of nano-filler addition on improving polyester matrix mechanical properties in dry and wet condition has been investigated.

The knowledge of the effects of moisture absorption and high temperature exposure on flexural, tensile and impact properties behaviour is not easily found in literature for hybrid polyester composites reinforced with HNT and MLG. This appears to be important with a view to broadening the industrial applications of these nano-composites in particular with reference to coating industry. The aim of making hybrid nanocomposites was to study whether synergistic effects can reduce the water degradation effect caused by water absorption at low weight fraction of 0.1 wt %.

## 2. Materials and Methods

**Figure 1:**
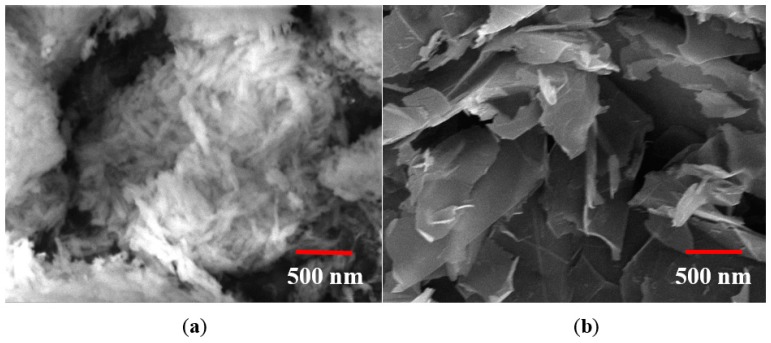
SEM images: (**a**) HNT; and (**b**) MLG.

HNT (Figure 1a) was used as a reinforcement filler and acquired from Sigma Aldrich (Irvine, UK). The diameter of HNT was between 30 and 70 nm with length 1–4 μm. It had a tube-like morphology with a density of 2.53 g/cm^3^ and a surface area 64 m^2^/g. HNT has low electrical and thermal conductivities and strong hydrogen interactions. The tubular morphology, high aspect ratio, and low percolation make HNT a prospective reinforcement for polyester and other polymers.

MLG (Figure 1b) of 12 nm average thickness and 4.5 μm average lateral size with a surface area of 80 m^2^/g was used as second filler, purchased from Graphene Supermarket. The polyester resin of the NORSODYNE O 12335 AL was acquired from East Coast Fibreglass, Newcastle, UK. The resin had a density of 1.2 g/cm^3^. The catalyst (hardener) was methyl ethyl ketone peroxide solution in dimethyl phthalate and purchased from East Coast Fibreglass, UK.

To produce monolithic polyester samples, the resin (Norsodyne O 12335 Al) was mixed with catalyst (Butanox M-50) in a polyester:catalyst ratio of 98:2. Following thorough hand mixing for 10 min, vacuum degassing was again carried out for 10 min. The resin was poured into silicone moulds (without any release agent) and cured at room temperature for 24 h, followed by post-curing at 80 °C for 2 h to ensure completion of the crosslinking [4,31]. In this research, four different samples were produced; monolithic polyester, 0.05 wt % HNT-0.05 wt % MLG, 0.1 wt % HNT, and 0.1 wt % MLG. Usually, there are different samples and the optimum concentration is related roughly with the mix with the best dispersion of fillers and minimum agglomeration. The viscosity of resins and characteristics of fillers also determine the dispersion quality. The concentration with optimum dispersion for different additives is difficult to quantify. For clay particles, optimum reinforcement is below 1 wt %. This is because dispersing higher weight fraction is difficult and agglomerated clay increases the liquid absorption and deteriorates the mechanical properties as the agglomerates act as stress concentration sites [32,33].

Ramanathan et al. revealed that PMMA poly(methyl methacrylate), when reinforced with 0.05 wt % of graphene sheets, can dramatically improve thermal and mechanical properties [34]. It was reported that the glass transition temperature increased by 30 °C [34]. We believe that the minimum concentration of 0.05 wt % is an ideal selection due to the significant effect, as revealed by other researchers [30,34].

## 3. Characterization

DMA (Model 8000, Perkin-Elmer, Waltham, MA, USA) was used to determine dynamic storage modulus (*E*′), and loss modulus (*E*″) of the samples. The loss factor tanδ was calculated as the ratio (E″/E′). Rectangular test specimens of dimensions 20 × 5 × 3 mm^3^ were used with a single cantilever clamp. All tests were carried out by a temperature sweep method (temperature ramp from 30 to 130 °C at 5 °C/min) at a constant frequency of 1 Hz. The maximum force of DMA was 10 N and applied during all DMA tests. The glass transition temperature (*T*_g_) was taken as the temperature value at the peak of tanδ curves. The densification of samples was calculated according to ASTM D792. The densities of polyester, hardener, HNT, and water were 1.2, 1.18, 2.53, and 0.9975 g/cm^3^, respectively. The following equations were used to obtain the experimental density and densification:

(1) $$\mathrm{Experimental}\;\mathrm{density}=\frac{\mathrm{weight}\;\mathrm{in}\;\mathrm{air}}{\mathrm{weight}\;\mathrm{in}\;\mathrm{air}-\mathrm{weight}\;\mathrm{in}\;\mathrm{water}}\;\times \;\mathrm{density}\;\mathrm{of}\;\mathrm{water}$$

(2) $$\mathrm{Densification}\;\left(\%\right)=\frac{\mathrm{experimental}\;\mathrm{density}}{\mathrm{theoretical}\;\mathrm{density}}\;\times \;100$$

To measure water absorption, rectangular specimens with dimensions 80 × 10 × 4 mm^3^ were immersed into the liquid media at room temperature. Before the weighing procedure, any retained water was removed from its surface with an absorbent paper. The water absorption in the sample was measured as percent weight increase in the samples. Equation (3) was used to calculate the water absorption in the specimens:

*W*_c_ = (*W*_t_ − *W*_o_) × 100/*W*_o_ (3)

where *W*_t_ is the weight of specimen at time *t* (i.e., after immersion in the liquid) and *W*_o_ is the initial weight of the sample, i.e., before placing in water. Light transmittance in UV-VIS spectroscopy (Shimadzu UV-2600, Kyoto, Japan) was used to quantify the dispersion of fillers in the polyester system. Tests were carried out on both dry and wet samples. Light transmittance of nano-filler dispersions in polyester was recorded at wavelength between 400 and 1400 nm. Five specimens of dimensions 80 × 10 × 3 mm^3^ were tested for each set of conditions. The optical transmittance graphs for dry and wet samples are also presented. The effect of water absorption on the mechanical properties of HNT-polyester nanocomposites was investigated after placing the specimens in water for 24 h at 60 °C and compared with the same nanocomposites in the dry condition (without immersion in water). The 60 °C immersion temperature is considered a moderate temperature in accelerating aging [35]. The temperature role was to accelerate the effects of water aging [13].

A Vickers microhardness test was performed using the Buehler Micromet II for the monolithic polyester and its nanocomposites. The load applied was 200 g for 10 s. After water immersion, the samples were taken out and the liquid completely wiped from the specimen surface.

**Figure 2:**
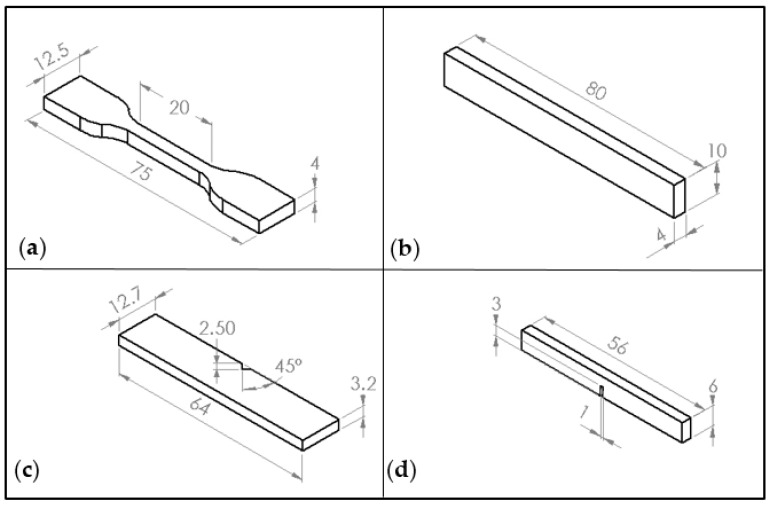
The illustration of specimens: (**a**) tensile; (**b**) flexural; (**c**) Charpy impact toughness; and (**d**) fracture toughness, *K*_1C_.

A tensile test and three-point bend (Figure 2a,b) tests were performed using an Instron Universal Testing Machine (Model 3382). Five specimens were tested for each composition. The displacement rate for the three-point bend and tensile tests were kept to 1 mm/min. Tensile test properties were carried out according to ISO 527 (Figure 2a) with a specimen thickness of 3 mm. The three-point bending test was performed according to ISO 178 with dimensions 80 × 10 × 4 mm^3^ (Figure 2b). A Charpy impact toughness test was performed according to ASTM D6110 (Figure 2c) using notch samples. AV-notch 45° was made in the centre of samples. The impact toughness was obtained using Equation (4), where *m* is the mass of hammer (kg), *g* is the standard gravity (9.81 m/s^2^), *h* is the length of hammer (m), and *t* is sample thickness (mm):

(4) $$\mathrm{Impact}\;\mathrm{toughness}=\frac{mgh\;(\cos\beta -cos\;\alpha )}{wt}$$

An Instron Universal Testing Machine (Instron, High Wycombe, UK) was also used to perform fracture toughness tests. The fracture toughness (*K*_1C_) was determined using a single edge notch three-point bending (SEN-TPB) specimen (ASTM D5045) as shown in Figure 2d. The displacement rate used was 1 mm/min. The dimensions were 3 × 6 × 36 mm^3^ with a crack length of 3 mm at the centre of the sample. *K*_1C_ was calculated using linear fracture mechanics by following relationship:

(5) $$K_{\mathrm{IC}}=\frac{P_{\max}\left(\frac{a}{w}\right)}{BW^{1/2}}$$

where *f (a/W)* is the calibration factor for the samples which is given as:

(6) $$f\left(\frac{a}{w}\right)=\frac{\left[\left(2+\frac{a}{w}\right)\left\{0.0866+4.64{\left(\frac{a}{w}\right)}^{2}+14.72{\left(\frac{a}{w}\right)}^{3}-5.6{\left(\frac{a}{w}\right)}^{4}\right\}\right]}{{\left(1-\frac{a}{w}\right)}^{3/2}}$$

An Alicona optical microscope was used to study the topographical features of the produced samples. The Alicona Infinite Focus optical microscope (G4, Alicona, Graz, Austria) was used to generate optical micrographs and measure topographical features. The Alicona optical microscope is a non-contact method (focus-follow method) for topography measurement. *R*_a_, *R*_q_, and *R*_z_ were obtained from the topography measurement. *R*_a_ is defined as the roughness average of the surfaces’ measured microscopic peaks and valleys [36]. *R*_q_ states the root mean square of the profile and is sensitive to surface variation [36]. *R*_z_ is defined as the highest and lowest point of the profile and is useful when products subject to stresses [36]. Scanning electron microscopy (SEM) analysis using a FEI Quanta 200 (FEI, Cambridge, UK), was carried out on the fractured surfaces of tensile specimens to evaluate the fracture modes in the samples. The fractured portions were cut from the specimens and a layer of gold was applied using an Emscope sputter coater, model SC500A (Quorum Technologies, East Sussex, UK).

## 4. Results

**Figure 3:**
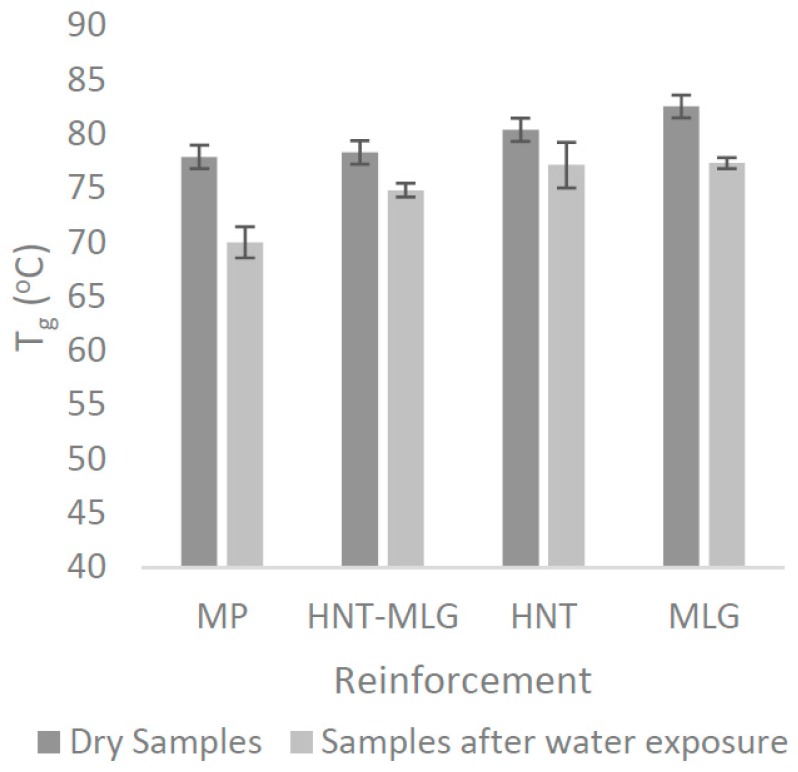
*T*_g_ of nanocomposites in dry conditions and after water exposure.

In this research, the addition of 0.1 wt % of HNT and MLG increased the *T*_g_ as shown in Figure 3. An increase in *T*_g_ with HNT and MLG shows that the fillers were uniformly dispersed [12,29]. As for HNT, the change in *T*_g_ associated with inorganic fillers was reported and proposed by others [37]. The two common factors were rigid phase reinforcement and destroying the epoxy-based polymer network structure [38]. Other authors also proposed that HNT and other clay particles restrict the mobility of polymer chains [39,40]. In case of MLG, when it is uniformly dispersed, the wrinkled texture and high surface area influence the maximum exothermic heat flow temperature by restricting polymer chain mobility, thereby causing an increase in *T*_g_ [30].

**Figure 4:**
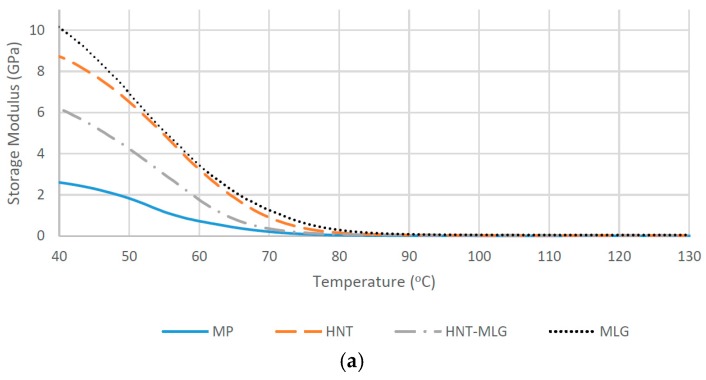

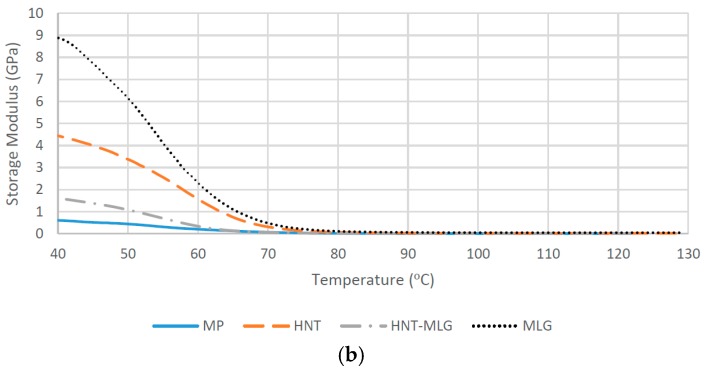
Storage modulus of nanocomposites in dry conditions (**a**) and after water exposure (**b**).

**Figure 5:**
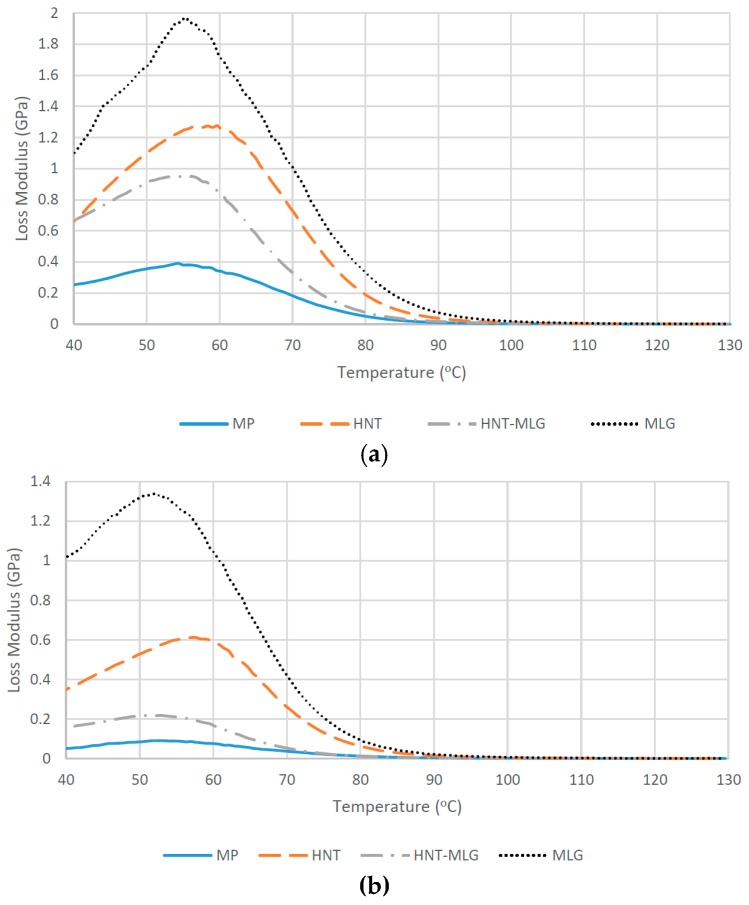
Loss modulus of nanocomposites in dry conditions (**a**) and after water exposure (**b**).

In dry conditions, monolithic polyester (MP) recorded the lowest value of *T*_g_ with 77.9 °C. The *T*_g_ increased to 78.3 °C for HNT-MLG polyester nanocomposites and increased to 80.4 °C in the case of 0.1 wt % HNT-reinforced polyester. The maximum *T*_g_ was in case of 0.1 wt % MLG-reinforced polyester with 82.6 °C (6% increase). After water exposure, the *T*_g_ decreased for all nanocomposites (compared to dry nanocomposites system). The lowest *T*_g_ was observed for MP as *T*_g_ dropped from 77.9 to 70 °C. The highest *T*_g_ was observed for 0.1 wt % MLG-reinforced polyester (77.34 °C). The lowering of *T*_g_ is an evidence of plasticization effect by water [41]. Moisture wicking along the fiber-matrix interface degrades the interfacial bond strength, resulting in loss of micro-structural integrity [41]. The storage modulus of nanocomposites for dry samples is shown in Figure 4a. The increase of the storage modulus at the glass transition temperature can be associated with the decrease in polymeric chain mobility [42] and the enhancement of stiffness [43]. In the case of 0.1 wt % HNT and 0.1 wt % MLG, there is a significant improvement of the storage modulus, particularly at lower temperature. The maximum storage modulus at 40 °C was recorded for the MLG filler. The storage modulus for all reinforced polyester later decreased as they approached the glass transition temperature (*T_g_*). It can be observed that the storage modulus increased while the loss modulus decreased for hybrid (0.05 wt % HNT-0.05 wt % MLG), 0.1 wt % HNT, and 0.1 wt % MLG-reinforced polyester compared with monolithic polyester. The storage modulus for nanocomposites exposed to water is shown in Figure 4b. It can be seen that the storage modulus and loss modulus (Figure 5) considerably decreased as a result of matrix softening [44].

**Figure 6:**
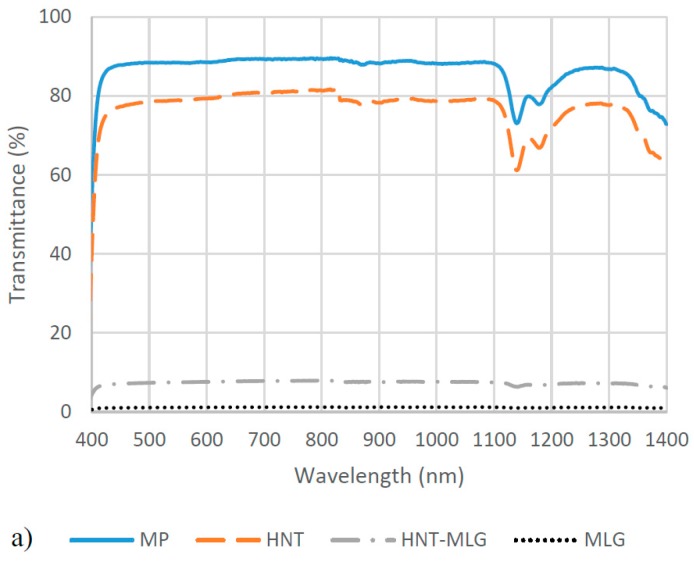

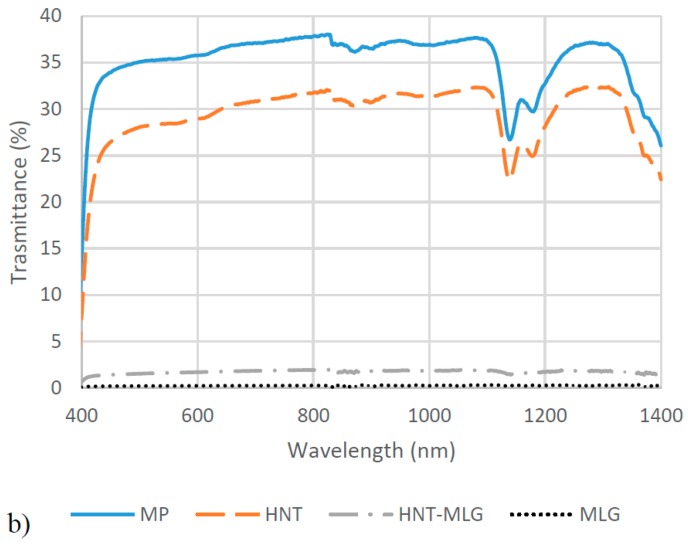
Optical transmittance of nanocomposites for dry conditions (**a**) and after water exposure (**b**).

The optical transmittance of the nanocomposites was investigated. In Figure 6a,b, it can be observed that MP is essentially highly transparent over the 400–1400 nm wavelength. The average transmittance value of MP is 72.9%. At 0.1 wt % HNT, it recorded 57.6% average value. The 0.05 wt % HNT-0.05 wt % MLG-reinforced polyester recorded only 4.3% optical transmittance. The 0.1 wt % MLG had an optical transmittance of 0.29%. It can be seen, even at 0.05 wt % HNT-0.05 wt % MLG, the optical transmittance dropped significantly. After water exposure, a similar trend was observed where monolithic polyester had the highest optical transmittance. However, the water exposure significantly reduced the optical transmittance for monolithic polyester (decrease of 46.3% compared to dry condition). The 0.1 wt % HNT-reinforced polyester lost 37% of optical transmittance due to water absorption. The optical transmittance for 0.05 wt % HNT-0.05 wt % MLG-reinforced polyester and 0.1 wt % MLG-reinforced polyester were also found to be decreased, but the values were not significant.

**Figure 7:**
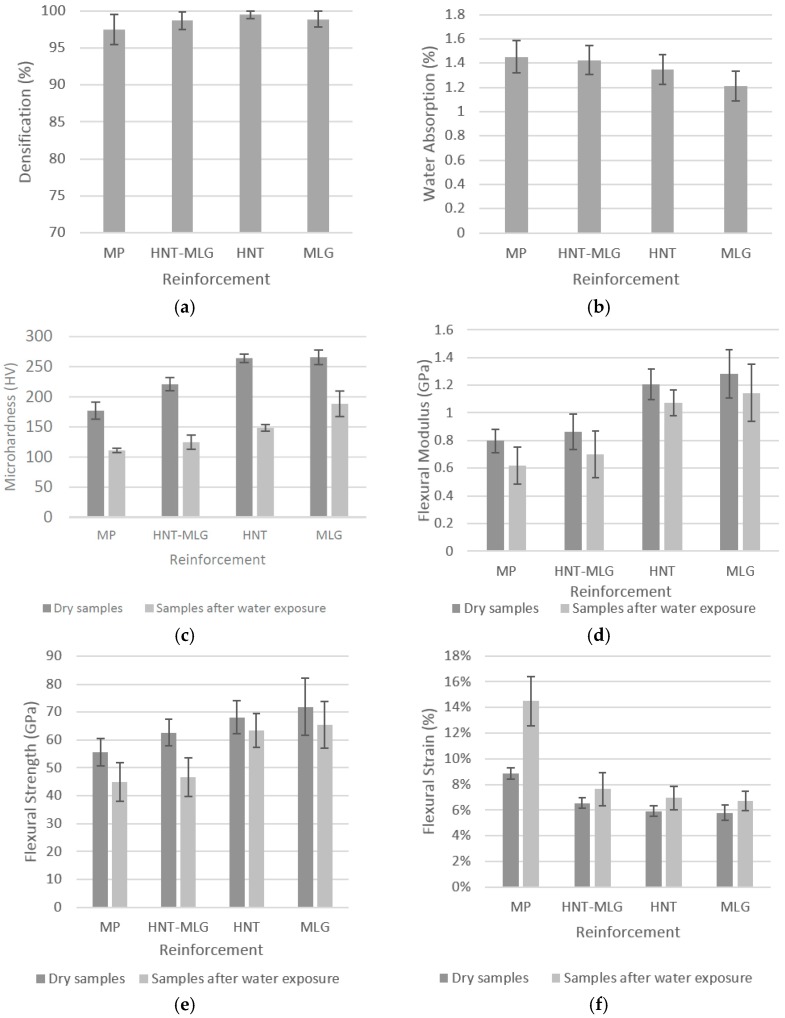

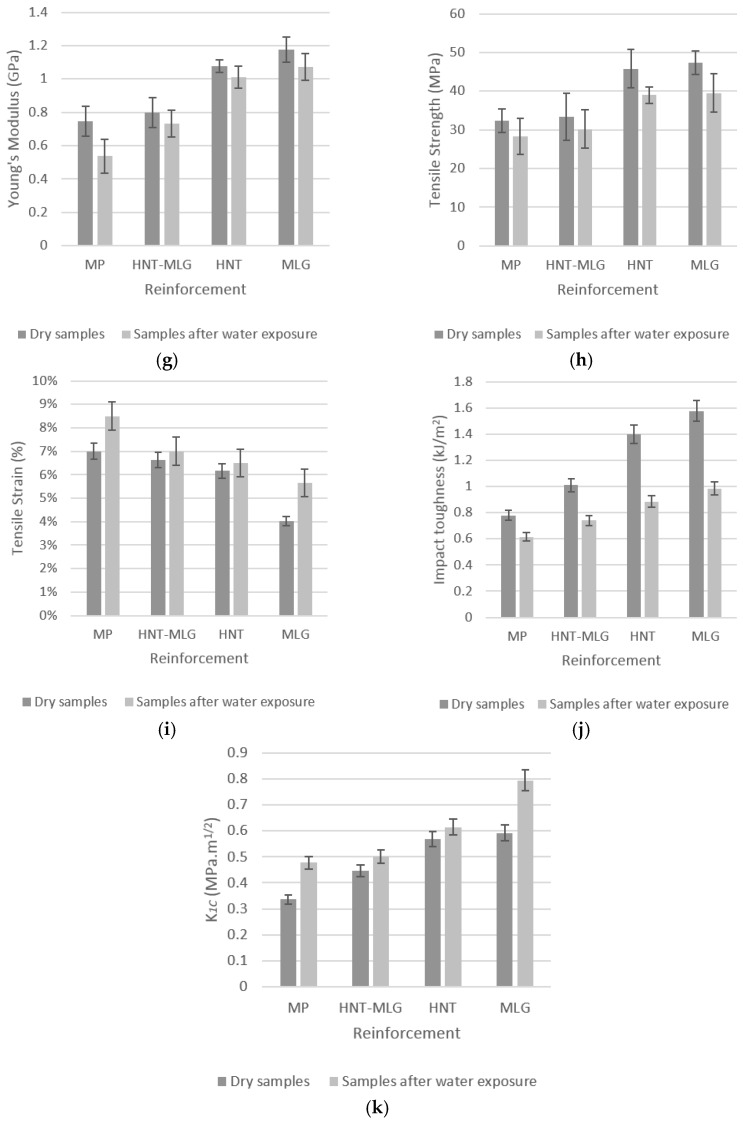
Mechanical properties of nanocomposites in dry conditions and after water exposure: (**a**) densification (%); (**b**) water absorption test (%); (**c**) microhardness (HV); (**d**) flexural modulus (MPa); (**e**) flexural strength (MPa); (**f**) flexural strain (%); (**g**) Young’s modulus (MPa); (**h**) tensile strength (MPa); (**i**) tensile strain (%); (**j**) impact toughness (kJ/m^2^); (**k**) *K*_1C_ (MPa.m^1/2^).

The densification of samples versus the type of reinforcement is shown in Figure 7a. The large standard deviations for monolithic polyester indicate porosity within the samples. That shows air entrapment during processing [45]. Another reason for this could be the quick curing of polyester resin as the volatiles could not escape during curing [46,47]. The casting technique used, on the other hand, is not usually considered 100% reproducible, like latex technology [30]. The water absorption test is shown in Figure 7b. It can be seen that the monolithic polyester absorbed more water than other nanocomposite systems. For 0.05 wt % HNT-0.05 wt % MLG-reinforced polyester recorded water absorption of 1.42%. The 0.1 wt % HNT-reinforced polyester recorded 1.35% and 0.1 wt % MLG-reinforced polyester with 1.2% of water absorption.

The microhardness result is shown in Figure 7c. Compared to monolithic polyester, the 0.05 wt % HNT-0.05 wt % MLG-reinforced polyester improved the microhardness from 177 to 221 HV (25% increase). The microhardness increased steadily in case of 0.1 wt % HNT (49% increase) and 0.1 wt % MLG (50.3% increase). After water exposure, the monolithic polyester recorded only 111 HV. The microhardness for nanocomposites exposed to water improved in case of 0.05 wt % HNT-MLG, 0.1 wt % HNT, and 0.1 wt % MLG, however, the values were lower than those in dry conditions. The reduction of microhardness was caused by the surface softening of polyester matrix by water [48,49]. Flexural modulus of the nanocomposites is shown in Figure 7d. For dry samples, the maximum flexural modulus was observed in case of 0.1 wt % MLG (60.6% increase) followed by 0.1 wt % HNT (increase 50.6%). For samples exposed in water, the similar trend was observed. The maximum flexural modulus was recorded in case of 0.1 wt % MLG. The flexural modulus increased from 0.62 to 1.15 GPa (increase 85.5%).

Flexural strength of nanocomposites in dry and wet conditions is presented in Figure 7e. Minimum flexural strength was recorded in the case of MP. The maximum flexural strength was observed in case of 0.1 wt % MLG-reinforced polyester. The flexural strength increased from 55.7 to 71.9 MPa (29% increase). After water exposure, the flexural strength showed degradation compared to unexposed samples. The lowest flexural strength was observed for MP with only 45 MPa. The flexural strength then steadily increased in case of 0.05 wt % HNT-0.05 wt % MLG (47 MPa), 0.1 wt % HNT (63 MPa) and 0.1 wt % MLG (65 MPa).

The variation in flexural strain of nanocomposites for dry and wet conditions is shown in Figure 7f. In comparison with MP, the flexural strain decreased with the incorporation of nano-fillers. The increase in strength and stiffness reduced the flexural strain. After water exposure, the flexural strain increased for all samples. This could be due to the fact that water fills the gaps between the fillers and polymer matrix, eventually leading to a decrease in flexural strength [50]. The water absorption leads to an increase of the plastic zone ahead of the crack, hence, increasing the flexural strain of all nanocomposites [51].

The variation of Young’s modulus is shown in Figure 7g. Monolithic polyester obtained a Young’s modulus of 0.75 GPa. The Young’s modulus increased 7% in case of 0.1 wt % HNT. The highest Young’s modulus was obtained for 0.1 wt % MLG reinforcement with an improvement of 60%. After water exposure, 0.1 wt % of MLG reinforcement also recorded the highest Young’s modulus with an increase of 98% compared to MP. The variation in tensile strength is shown in Figure 7h. At 0.1 wt % MLG reinforcement, the highest tensile strength was observed. The tensile strength increased from 32.4 up to 47.3 MPa (46% increase) for dry samples. As for wet samples, the tensile strength increase from 28.3 to 39.5 MPa. The tensile strain graph is shown in Figure 7i. In dry conditions, the tensile strain tends to have a lower value than samples exposed in a wet environment. Dry samples were stiffer and have higher strength than samples tested after water exposure.

The variation of impact toughness is shown in Figure 7j. For dry samples, MP recorded a value of 0.78 kJ/m^2^. In the case of 0.05 wt % HNT-0.05 wt % MLG, the impact toughness increased to 1 kJ/m^2^. A further increase of impact toughness can also be seen for samples reinforced with 0.1 wt % HNT (1.4 kJ/m^2^). The maximum increase of impact toughness was seen for samples reinforced with 0.1 wt % MLG (1.6 kJ/m^2^). The fracture toughness (*K*_1C_) is shown in Figure 7k. The maximum fracture toughness was observed in the case of 0.1 wt % MLG reinforcement. The fracture toughness of this polyester system has been enhanced with the addition of 0.05 wt % HNT-0.05 wt % MLG, 0.1 wt % HNT, and 0.1 wt % MLG. The fracture toughness increased from 0.3 to 0.6 MPa.m^1/2^ (100% increase). In general, the water exposure increased the fracture toughness of the nanocomposites. Polyester reinforced with 0.1 wt % MLG recorded the highest fracture toughness. The fracture toughness increased from 0.48 to 0.8 MPa.m^1/2^ (67% increase).

In general, the addition of HNT-MLG, HNT, and MLG improved the mechanical properties both in dry conditions, and after water immersion exposure. The improvement depends on several aspects, such as dispersion and interfacial interaction. When HNT was blended in an unsaturated polyester matrix, the stress transferred from the polymer to the mineral, hence, the increased mechanical properties of the composites [52]. MLG, on the other hand, displays outstanding properties due to their high aspect ratio and high mechanical strength [52]. It was also observed that MLG is more effective than nanoclay in improving the mechanical properties of unsaturated polyester. The gradual and smoother failure of the monolithic polyester took place at lower load levels than the HNT-MLG system. Higher load levels took place in the case of MLG-reinforced polyester, indicating an earlier stabilization in terms of tensile and flexural strain [53]. It was clearly observed that synergistic effects are not effective at the low concentration of 0.1 wt % to produce remarkable improvement in mechanical properties of produced nanocomposites.

### 4.1. Topographic Profile

**Figure 8:**
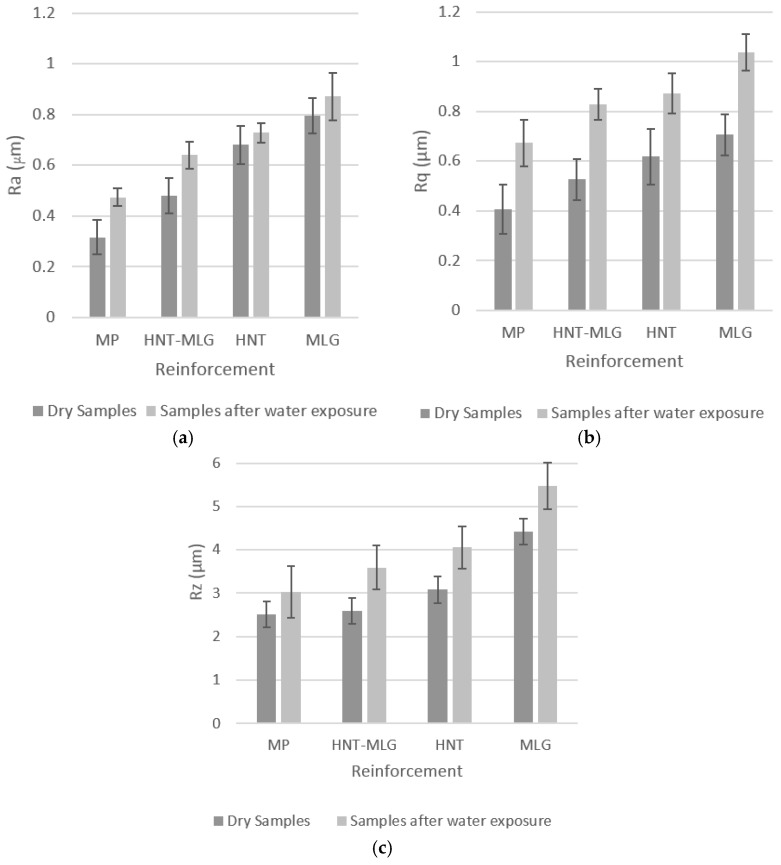
Topographic features of nanocomposites in dry conditions and after water exposure: (**a**) average of surface’s roughness, *R*_a_ (µm); (**b**) root mean square of profile, *R*_q_ (µm); (**c**) highest and lowest point of the profile, *R*_z_ (µm)

The *R*_a_ (Figure 8a) value for monolithic polyester was 0.32 μm. The *R*_a_ then increased steadily for samples reinforced with 0.05 wt % HNT-MLG (0.48 μm), 0.1 wt % HNT (0.68 μm), and 0.1 wt % MLG (0.8 μm). The *R*_a_ values for samples exposed in water increased compared to the dry condition. The water molecules diffused through the polymer matrix and congregated around the particles [54]. This led to an increase in the plastic zone, which then increased the surface roughness of the nanocomposites.

A similar trend was also observed for *R*_q_ and *R*_z_ for nanocomposites tested in dry conditions and after water exposure. *R*_q_ is the root mean square of the profile and sensitive to surface variation 29. The peak-to-valley heights or *R*_z_ measures the highest and lowest point of the profile. *R*_q_ for 0.1 wt % MLG-reinforced polyester in air was 0.71 µm and the *R*_z_ was 4.43 µm. After water exposure the maximum *R*_q_ and *R*_z_ for 0.1 wt % MLG-reinforced polyester was 1 and 5.5 μm. A high value of *R*_a_ (with low *R*_z_ value) can be on indicator of smoother samples surfaces, absence of agglomerates and uniform dispersion of nano-fillers. A low value of *R*_a_, but high *R*_z_, value shows the existence of deep surface notches, agglomerates, and non-uniform dispersion of nano-fillers. In general, *R*_q_ (Figure 8b) and *R*_z_ (Figure 8c) were lower in the dry condition and higher after water exposure. After water exposure, a coarser topography was observed.

**Figure 9:**
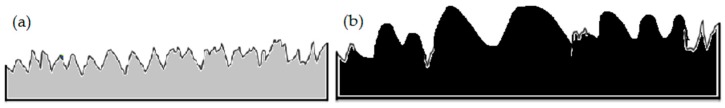
Topography profile of samples in dry conditions (**a**) and after water exposure (**b**).

**Figure 10:**
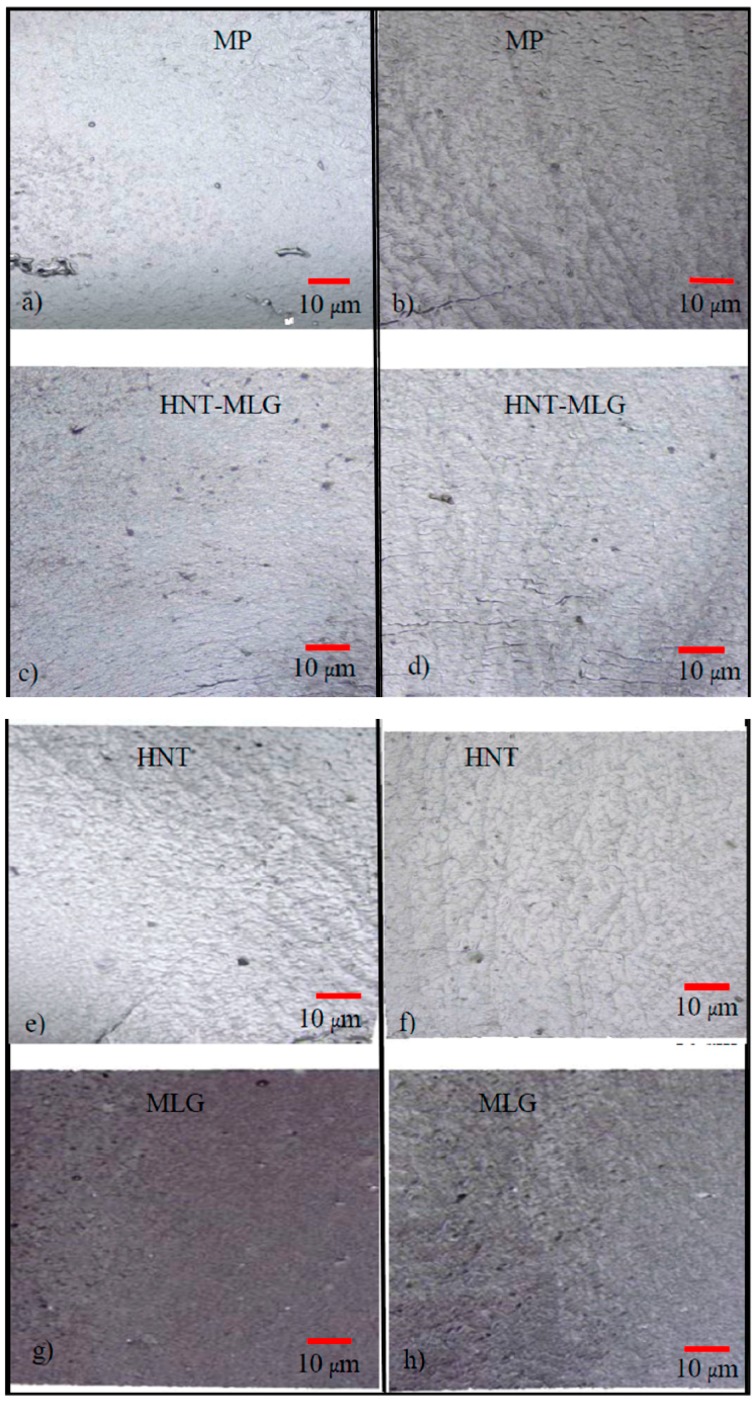
Surface profile of nanocomposites before and after water exposure: (**a**) monolithic polyester before water exposure; (**b**) monolithic polyester after water exposure; (**c**) HNT-MLG nanocomposites before water exposure; (**d**) HNT-MLG nanocomposites after water exposure; (**e**) HNT nanocomposites before water exposure; (**f**) HNT nanocomposites after water exposure; (**g**) MLG nanocomposites before water exposure; (**h**) MLG nanocomposites after water exposure.

The flexural strain samples (from three point-bend test) used for the surface roughness measurement kept increasing with the coarser topography. This can be attributed to lower stiffness and strength values. A schematic illustration on topography difference between polyester nanocomposites tested in air and after water exposure is illustrated in Figure 9. The topography profile after water exposure became coarser because of the plasticization effect. The increase of high peaks can be linked to the water absorption. The topography surface profile of nanocomposites for samples tested in air and wet conditions is presented in Figure 10. It can be observed that after water exposure, the surface profiles were coarser than dry samples.

### 4.2. Fractography Analysis

**Figure 11:**
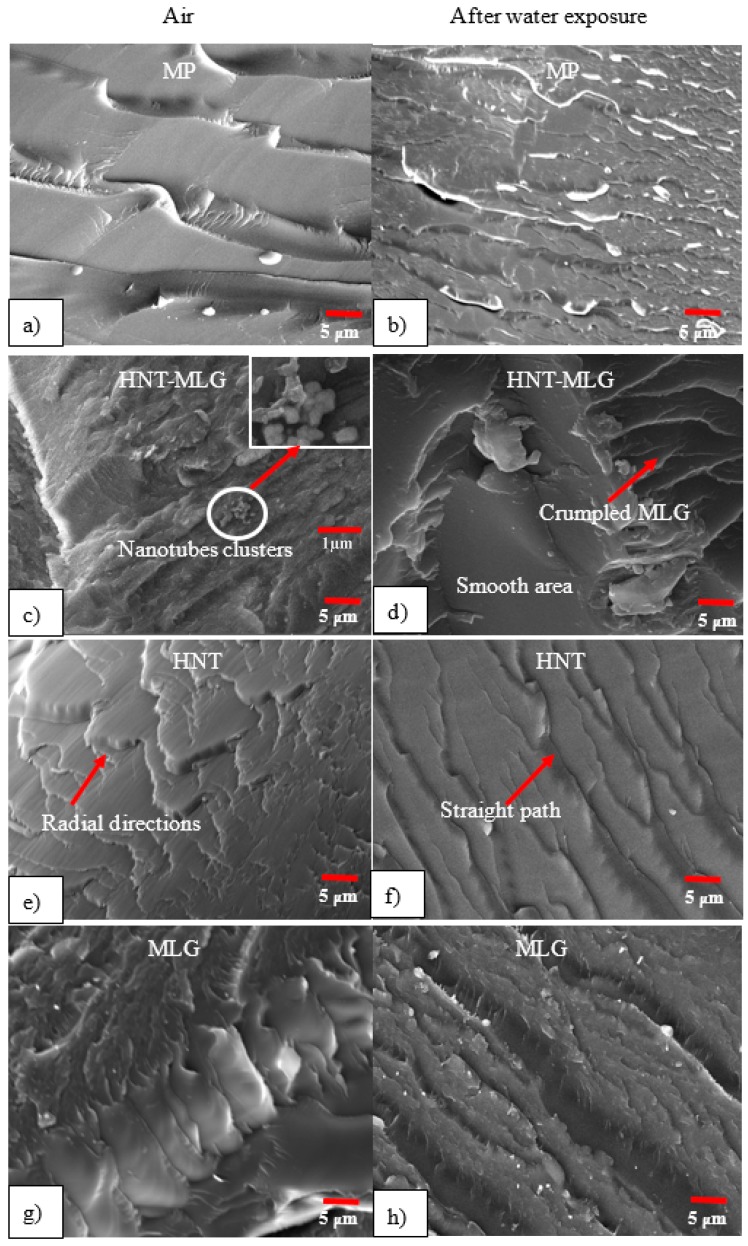
SEM images of nanocomposites before and after water exposure: (**a**) monolithic polyester before water exposure; (**b**) monolithic polyester after water exposure; (**c**) HNT-MLG nanocomposites before water exposure; (**d**) HNT-MLG nanocomposites after water exposure; (**e**) HNT nanocomposites before water exposure; (**f**) HNT nanocomposites after water exposure; (**g**) MLG nanocomposites before water exposure; (**h**) MLG nanocomposites after water exposure.

The SEM images of fractured surfaces are shown in Figure 11. As the cracks propagate, material is lost most likely in the form of round particles, as can be observed in Figure 11a. The image also revealed that the monolithic sample was showing river markings, which can be associated with a fast, brittle fracture mode [55]. It is evident that there are no crack bridging mechanisms available in monolithic polyester. When the cracks propagated, they move with less diversions. After water exposure, the monolithic polyester shows a smoother surface with weaker crack lines. The de-bonding for MP is in the form of long and straight lines.

Synergistic effects are not effective at a concentration of 0.1 wt % to produce considerable improvement in mechanical properties of produced hybrid nanocomposites [56]. De-bonded clusters of fillers from polyester matrix can be seen for hybrid sample (Figure 11c). The size of the clusters is relatively small with considerably small spacing. The material in the vicinity of the clusters and the distance between them may not have a significant effect in mechanical properties [57].

The effect of MLG was also noticeable on the surface of the 0.05 wt % HNT-0.05 wt % MLG nanocomposites. The crumpled structure of MLG is shown in Figure 11d. The effect of water on hybrid nanocomposites suggest that surface roughness was reduced compared to samples tested in dry conditions [58]. De-bonding and pull-out of fillers were observed in hybrid samples, which are responsible for the moderate toughening in Figure 11d [59]. For the HNT samples, the interlocking effect can alter the crack formation mechanism. In Figure 11e, the crack started from a defect point and emanated radially. A similar phenomenon has been reported elsewhere [55]. Crack lines are straighter after the HNT-reinforced polyester samples were exposed to water. The nanocomposites containing HNT particles showed a plasticization effect where the crack propagation became easier and faster. Graphene-based materials are often compliant, and when dispersed in a polymer matrix, they are typically not observed as rigid discs, but rather as bent or crumpled platelets [60]. The wrinkle structure of MLG has better interfacial interactions than the tubular structure of HNT [61]. The wrinkled structure significantly improves the interfacial interactions with the polyester chains. Figure 11g shows no particular crack orientation. This is because MLG has the ability to prevent the propagation of cracks and cracks detour around the MLG to proceed [62]. After water exposure, micro-cracks and pronounced river markings can be observed for the MLG-reinforced sample. It is evident that presence of HNT and MLG fillers increased the fracture surface roughness. That is an indicator of crack deflection mechanism, which increases the absorbed energy of fractures by increasing the crack length during deformation [52].

The fracture nature between monolithic polyester, hybrid, HNT-, and MLG-reinforced polyester are different from each other. Lower resistance to crack propagation shows more straight paths and a smooth surface. This can be observed in the case of monolithic polyester and hybrid nanocomposites. Hybrid nanocomposites showed a moderate toughening mechanism, but slightly better than unfilled polyester. It can be observed that 0.1 wt % HNT-reinforced polyester shows high resistance to crack propagation compared to monolithic polyester and hybrid nanocomposites with round-ended cracks. The high aspect ratio of MLG, however, showed superior toughening than other nanocomposite systems. The force required for crack propagation of 0.1 wt % MLG-reinforced polyester was higher based on the SEM images.

Results obtained suggested that there was no significant improvement in the water barrier properties and mechanical performance in hybrid nanocomposites. This is because 0.05 wt % of HNT and MLG are either not enough to produce significant synergistic effects or there are no synergistic effects between HNT and MLG. Based on SEM images, there was no evidence that HNT and MLG were poorly dispersed. The weight fraction used was only 0.1% and, therefore, the inferior dispersion state can be ruled out as a cause for the degradation of mechanical properties. It is noted from the literature that the diffusion of moisture can be distributed throughout the polymer matrix or be drawn to form water clusters [63]. In this research, the formation of water clusters was not observed in SEM images, but a plasticization effect was clearly noticed. Therefore, plasticization of the matrix is mainly responsible for the degradation of mechanical properties for all nanocomposite systems (MP, HNT-MLG, HNT-, and MLG-reinforced polyesters).

## 5. Conclusions

The effect of short water absorption with 60 °C temperature on the mechanical properties of HNT (halloysite nanotube)- and multi-layer graphene (MLG)-reinforced polyester has been studied due to a strong motivation to identify low-cost methods for improving the barrier performance of polymers. It is shown that polyester matrix is vulnerable to water exposure. Addition of small amounts of HNT and MLG decreased the weight gain of the nanocomposites compared to monolithic polyester. MLG-reinforced polyester showed superior strength compared to hybrid and HNT in dry conditions and after water exposure. It can be observed that graphene-based materials show great promise for the next generation of environmental barrier materials. The SEM images revealed fewer cracks for all samples exposed to water. Nano-filler and matrix interface weakening were the main failure mechanisms induced by water exposure. The degradation of mechanical properties related to water absorption caused softening of the polymer matrix, which lowered the strength of the nanocomposites. Fracture toughness of nanocomposites after water exposure increased because of the plasticization effect. The surface roughness of all nanocomposite systems increased after water exposure. This can be attributed to the high peaks and plasticized crack zone, which then produced a coarser topography. This study provided evidence that the synergistic effect of HNT-MLG hybrid nanocomposites at low content (0.05 wt % HNT-0.05 wt % MLG) was insufficient to produce remarkable mechanical properties under dry conditions and after water exposure. More research should be conducted to improve the mechanical properties of hybrid composites exposed to different liquid environments at different temperatures.
